# Antipsychotic use in dementia: the relationship between neuropsychiatric symptom profiles and adverse outcomes

**DOI:** 10.1007/s10654-020-00643-2

**Published:** 2020-05-15

**Authors:** Christoph Mueller, Christeena John, Gayan Perera, Dag Aarsland, Clive Ballard, Robert Stewart

**Affiliations:** 1 King’s College London, Institute of Psychiatry, Psychology and Neuroscience, De Crespigny Park, London, UK; 2 South London and Maudsley NHS Foundation Trust, London, UK; 3 Stavanger University Hospital, Centre for Age-Related Disease, Stavanger, Norway; 4 University of Exeter Medical School, Exeter, UK

**Keywords:** Dementia, Antipsychotics, Risk stratification, Prognosis, Mortality, Stroke

## Abstract

Antipsychotic treatments are associated with safety concerns in people with dementia. The authors aimed to investigate whether risk of adverse outcomes related to antipsychotic prescribing differed according to major neuropsychiatric syndromes—specifically psychosis, agitation, or a combination. A cohort of 10,106 patients with a diagnosis of dementia was assembled from a large dementia care database in South East London. Neuropsychiatric symptoms closest to first dementia diagnosis were determined according to the Health of the Nation Outcome Scales’ mental and behavioural problem scores and the sample was divided into four groups: ‘agitation and psychosis’, ‘agitation, but no psychosis’, ‘psychosis, but no agitation’, and ‘neither psychosis nor agitation’. Antipsychotic prescription in a one-year window around first dementia diagnosis was ascertained as exposure variable through natural language processing from free text. Cox regression models were used to analyse associations of antipsychotic prescription with all-cause and stroke-specific mortality, emergency hospitalisation and hospitalised stroke adjusting for sixteen potential confounders including demographics, cognition, functioning, as well as physical and mental health. Only in the group ‘psychosis, but no agitation’ (n = 579), 30% of whom were prescribed an antipsychotic, a significant antipsychotic-associated increased risk of hospitalised stroke was present after adjustment (adjusted hazard ratio (HR) 2.16; 95% confidence interval (CI) 1.09–4.25). An increased antipsychotic-related all-cause (adjusted HR 1.14; 95% CI 1.04–1.24) and stroke-specific mortality risk (adjusted HR 1.28; 95% CI 1.01–1.63) was detected in the whole sample, but no interaction between the strata and antipsychotic-related mortality. In conclusion, the adverse effects of antipsychotics in dementia are complex. Stroke risk may be highest when used in patients presenting with psychosis without agitation, indicating the need for novel interventions for this group.

**Electronic supplementary material:**

The online version of this article (10.1007/s10654-020-00643-2) contains supplementary material, which is available to authorized users.

## Introduction

Neuropsychiatric symptoms, such as agitation and psychosis, are common and highly impactful complications of dementia [1] and are major determinants of poor quality of life, carer burden and healthcare costs. They are also associated with more rapid dementia progression and increased mortality [2, 3]. Antipsychotic medications are often considered in the management of these symptoms; however, while meta-analyses indicate significant benefits, these are small, with Cohen’s *d* effect sizes lower than 0.2 across trials of antipsychotics to treat psychosis in Alzheimer’s disease [4–7].

These very modest benefits have to be balanced against potential harms, and concerns have been raised around an increased risk of mortality, cerebrovascular events and hastened cognitive decline in patients with dementia prescribed these agents [8, 9]. The US Food and Drug Administration has issued a black box warning against the use of antipsychotic medication in dementia related psychosis and has not approved any antipsychotic medication for treatment of aggression in dementia [6]. Conversely, the European Medicines Agency has approved risperidone as the only antipsychotic for the short-term treatment of persistent aggression in patients with moderate to severe Alzheimer’s dementia who are not responsive to non-pharmacological interventions and who pose a risk of harm to self or others [10].

Agitation and psychosis are nonetheless frequently interlinked in dementia [11] and there is an increasing recognition that patient characteristics, especially the presence of certain neuropsychiatric symptoms, may be strong influencers of risks related to antipsychotic prescribing [3, 12]. Using routinely collected and clinician-rated ‘real-world’ data on the hyperactivity and psychosis clusters of neuropsychiatric symptoms in dementia [13], we aimed to investigate whether antipsychotic medications pose different hazards for adverse health outcomes in stratified analyses, in order to evaluate the potential for a more targeted approach to antipsychotic prescribing in dementia. The presence of additional cerebral pathologies in psychosis in dementia [14–17] suggests higher antipsychotic hazards in this group, but a substantial proportion of patients with psychosis in dementia are not distressed or agitated [18]. As neuropsychiatric symptoms have been reported as potentially stronger correlates of adverse outcomes than their treatments [19] (with agitation possibly yielding a higher risk than psychosis [3]) we hypothesized that patients with psychosis only might be at an increased risk of antipsychotic-related adverse outcomes, while in other groups addressing the impact of the distress/aggression itself might partly counteract the adverse effects of antipsychotics. As secondary outcomes we evaluated whether risks differed in relation to dementia subtype diagnosis or specific antipsychotic prescribed.

## Methods

### Data source

Data for this study were assembled using the South London and Maudsley NHS Foundation Trust (SLaM) Clinical Record Interactive Search (CRIS) platform. SLaM serves a population of over 1.36 million residents, across four South London boroughs (Lambeth, Lewisham, Southwark, and Croydon) and is one of Europe’s largest healthcare providers for dementia and mental illness. Since 2006 all services in SLaM have adopted fully-electronic health records to enhance confidential and efficient storage of information. CRIS provides research access to over 400,000 anonymized health records from SLaM within a robust governance framework [20, 21] and has received ethical approval as an anonymized data resource (Oxford Research Ethics Committee C, reference 08/H0606/71+5).

We extracted data from structured fields routinely completed in the source record and from clinical documents. Identification of relevant information from free-text records was conducted through bespoke natural language (NLP) processing algorithms using the General Architecture for Text Engineering (GATE) software [22]. See supplementary document for a detailed description of the NLP algorithms applied to ascertain antipsychotic prescription, diagnosis and Mini-mental State Examination Score (MMSE) [23]. Further, CRIS has been linked to national data on hospitalisations (Hospital Episode Statistics (HES)) [24] and Office of National Statistics (ONS) death certificate data, enabling relevant health outcome data (mortality, hospitalisation) to be extracted for the current analyses.

### Sample and stratification

We included patients who received a first diagnosis of dementia in SLaM services between 1st January 2007 and 31st December 2015. Patients were excluded if they had a history of psychotic disorder prior to onset of dementia (as in this population antipsychotics are prescribed for functional psychosis rather than dementia), if they were diagnosed with a Lewy body dementia (as these pathologies inherently carry higher antipsychotic risks), and if they made use of acute hospital psychiatric liaison services in a six months (3 months either side) window around dementia diagnosis (as these patients tend to have higher levels of co-morbidities and are more likely to be subject to short-term use of antipsychotics for delirium episodes).

To stratify the sample according to neuropsychiatric symptoms present at the time of dementia diagnosis, we used the Health of the Nation Outcome Scales (HoNOS) [25], whereby the score recorded closest to first dementia diagnosis was applied. The HoNOS is a validated and well-established measure of patient wellbeing commonly used in UK mental health and dementia services and comprises 12 clinician-rated subscales. Each subscale is rated from 0 (no problem) to 4 (severe or very severe problem). For ease of interpretation, the scores were dichotomised to ‘minor or no problems’ (scores 0 or 1) and ‘mild to severe problems’ (scores 2–4). ‘Agitation’ was defined on the basis of a score of at least two or more on the HoNOS ‘behavioural disturbance’ scale, and ‘psychosis’ on the basis of a score of two or more on the HoNOS ‘problems associated with hallucinations and/or delusions’ scale. Both items are associated with an increased all-cause mortality risk in populations of people with dementia [26, 27].

From this information, we created four groups: ‘agitation and psychosis’ (Ag+P+), ‘psychosis, but no agitation’ (Ag–P +), ‘agitation, but no psychosis’ (Ag+P–), and ‘neither agitation nor psychosis’ (Ag–P–).

To achieve one of our secondary objectives, we stratified the sample according to dementia diagnosis subtype. The dementia subtype diagnosis was classified according to ICD-10 [28] through NLP supported by structured fields in the source record as follows: Alzheimer’s disease (F00.0 and F00.1), vascular dementia (F01), mixed-type dementia (including Alzheimer’s disease and vascular dementia or F00.2) and other/unspecified dementia (F03 or no subtype mentioned).

### Exposure and outcome variables

GATE-supported natural language processing algorithms on CRIS include one which ascertains pharmacotherapy from a comprehensive gazetteer of all past and current generic medication names (and most trade names); this was used to identify patients recorded as taking antipsychotic medications in a one year window (6 months either side) around first dementia diagnosis.

This medications application is designed to extract the names of medications that are inferred as currently prescribed to the patient [29, 30] and is described in more detail in the accompanying supplementary document, including examples of text captured. The application was developed through expert annotation, whereby domain experts coded whether the medication prescription was present either based on their expert experience and/or pre-defined coding rules. Negation statements (e.g. ‘not using any antipsychotic’) are not specifically ascertained as a group, but are subsumed in the wider category of non-relevant statements [21]. The application preferentially detects medications with corresponding dosage information, or where present use is explicitly mentioned (e.g. ‘currently on’) or is discernible through inference (e.g. ‘patient’ is faring better on risperidone). The precision for current use of antipsychotic medication across CRIS was found to be 81% and the recall of ever use of antipsychotic medication 77%.

First, all medications in chapter 4.2. (Central nervous system/Drugs used in psychoses and related disorders) of the British National Formulary [31] were considered as exposures. To examine whether specific antipsychotics differed in their hazard profile we further determined whether patients were prescribed risperidone, olanzapine, quetiapine, any second-generation antipsychotic or any first-generation antipsychotic.

In the whole cohort and aforementioned strata (neuropsychiatric symptoms/subtype diagnoses) patients receiving any antipsychotic were compared to non-receivers in relation to adverse health outcomes. We established the time to events for each patient for the following four outcomes: (1) all-cause mortality, (2) any emergency hospitalisation to acute care (non-psychiatric) hospitals [24], (3) hospitalisation due to stroke, and (4) stroke-specific mortality. Hospitalised stroke was defined on the basis of ICD-10 codes [28]. As the narrow definition of stroke (ICD-10 codes I61, I63, and I64) usually applied to national hospitalisation (HES) data [32] might miss a substantial proportion of confirmed stroke cases and misclassify a considerable number of suspected strokes [33], we applied a wider definition identifying hospitalisations in which I60 to I67 (Cerebrovascular diseases section) and G45 (Transient cerebral ischaemic attacks and related syndromes) were recorded as primary diagnosis in discharge documentation. We only considered the primary diagnosis to ensure that the stroke was a new event and the main cause for the hospitalisation. A patient was considered to have died of a stroke if the aforementioned ICD-10 codes were listed as underlying or primary cause of death on ONS death certificate data. Mortality data was available to a census point on 10th December 2016 and hospitalisation data until a census point on 31st March 2016.

### Covariates

From structured fields and supported by natural language processing applications we ascertained socio-demographic factors (age, gender, marital status, ethnicity, and a neighbourhood-level index of multiple deprivation [34]), level of cognitive impairment (identified via MMSE score closest to the date of diagnosis [23]), and whether the patients had been hospitalised in the year before dementia diagnosis. In addition, we extracted data from the relevant remaining HoNOS subscales.

### Statistical analyses

STATA 13 [35] was used for all analyses. Descriptive statistics were generated and presented accordingly. We constructed three Cox regression models to examine whether exposure to antipsychotics was related to the adverse outcomes (hospitalisation, stroke, all-cause mortality, stroke-specific mortality). Model 1 was adjusted for age and gender. Model 2 included age, gender, marital status, ethnicity, index of deprivation, MMSE score and dementia subtype (except in the analysis stratified for dementia subtype). In Model 3 the items from Model 2 were included and we added HoNOS subscales (excluding agitation and psychosis when we stratified for these variables) and previous hospitalisation.

First, we examined antipsychotic risks in the whole sample and then in the aforementioned strata (neuropsychiatric symptoms/subtype diagnoses). We further performed separate analyses for each individual subgroup (symptom profile/subtype diagnosis), comparing the HR in the subgroup to the HR in all other subgroups combined by including an interaction term (treatment*subgroup) in Model 3. As the power of the test for interaction is lower compared to the test of direct effects, we followed the recommendation to raise the type one error rate to increase power [36]. We elected to increase the error rate from 5% to 10% and considered interactions with *p* < 0.1 as true interactions. Lastly, again using the full sample, different antipsychotic medications were compared in relation to hazardous outcomes.

Of patients with HoNOS scores on agitation and psychosis available at dementia diagnosis, 18% had missing data on at least one of the other covariates (most commonly MMSE (10%) or marital status (4%) were the only missing variables). As we judged missingness in this sample to be random, we imputed missing values using chained equations to maximise statistical power [37]. Using the *mi* package in STATA we created 20 imputed datasets through replacing missing values through simulated values assembled from covariates and outcome values. Rubin’s rules [38] were applied to combine coefficients in final analyses.

## Results

**Fig. 1:**
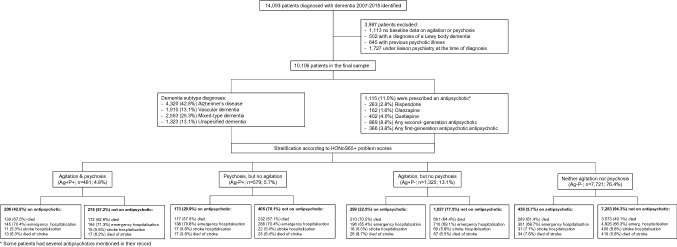
Flow chart

We identified 14,093 patients diagnosed with dementia in SLaM services between 2007 and 2015. Of those, 1113 patients were excluded as no baseline data was available on HoNOS ratings of agitation or psychosis, 502 patients with a diagnosis of a Lewy body dementia, 645 with a previous psychotic illness and 1727 patients as they made use of liaison psychiatry services at the time of diagnosis. The final sample consisted of 10,106 patients with a mean age at diagnosis of 81.1 (SD 8.7) years. Mean MMSE score at diagnosis was 18.7 (SD 6.4), 6384 (63.2%) patients were female and 1115 (11.0%) were prescribed an antipsychotic around the time of dementia diagnosis. See Fig. 1 for a flow-chart of cohort composition and outcomes.

In total 4320 (42.8%) patients had Alzheimer’s disease (AD) recorded as diagnosis, 2553 (25.3%) had mixed-type dementia (Alzheimer’s disease and vascular dementia), 1910 (18.9%) had vascular dementia, and 1323 (13.1%) were diagnosed as having other or unspecified dementia. Antipsychotic prescribing at the time of dementia diagnosis was most prevalent in those with other/unspecified dementia (17.9%), followed by vascular dementia (15.4%) and was least likely in those with Alzheimer’s disease (8.4% in pure AD and 8.7% in mixed-type dementia).

### Adverse outcomes associated with antipsychotic prescribing in the whole sample

Of the whole cohort, 5373 (53.2%) patients died in the follow-up period with a median survival time of 4.29 years (interquartile range 2.02–7.60 years). Moreover, 6797 (67.3%) had at least one emergency hospitalisation, 670 (6.6%) were recorded as having a hospitalised stroke and 619 (6.1%) died of a stroke according to their death certificate. In Cox regression models adjusting for age, gender, ethnicity, marital status, MMSE, deprivation score and dementia subtype (Model 2), antipsychotic prescription was related to an increased risk of all-cause mortality (hazard ratio (HR) 1.22; 95% CI 1.12–1.32) and stroke-specific mortality (HR 1.27; 95% CI 1.02–1.60), but not for emergency hospitalisation (HR 1.07; 95% CI 0.99–1.15) or hospitalised stroke (HR 1.02; 95% CI 0.80–1.29). After further adjustment for HoNOS scores and previous hospitalisation (Model 3), an increased all-cause mortality risk (HR 1.14; 95% CI 1.05–1.24) and stroke-specific mortality risk (HR 1.28; 95% CI 1.01–1.63) remained, but no significant effects were detected in relation to emergency hospitalisation or stroke (see Table 4 for Model 3 and Supplementary Table 1 for results of all models).

### Characteristics of the sample stratified according to neuropsychiatric symptom profile

In our sample 23.6% of patients presented with hyperactivity and psychosis neuropsychiatric symptoms at the time of dementia diagnosis; whereby agitation was present in 17.9% and psychosis in 10.5% of the sample. According to our group definitions, 481 (4.8%) presented with ‘agitation and psychosis’ (Ag+P+), 579 (5.7%) with ‘psychosis, but no agitation’ (Ag–P +), 1325 (13.1%) with ‘agitation, but no psychosis’ (Ag+P–) and the remaining 7721 patients with neither of the two neuropsychiatric symptoms (Ag–P–).

Sample characteristics and comparisons between groups are presented in Table 1. In comparison to those without either symptom, patients with agitation and/or psychosis had a lower MMSE at diagnosis, lived in more deprived neighbourhoods, were less likely to have a diagnosis of Alzheimer’s disease and more likely to be diagnosed with vascular or unspecified dementia, showed an increased occurrence of depressed mood, as well as physical health and functional problems. Amongst those with neuropsychiatric symptoms, patients with ‘psychosis, but no agitation’ (Ag–P +) were more likely to be female, less likely to be married or cohabiting, had a higher MMSE score, were less likely to have substance use, self-harm, physical health or functional problems (with the exception of living conditions). The Ag+P+ group included the highest proportion of patients prescribed antipsychotics (42.8%), followed by 29.9% in the Ag–P + group and 22.5% in Ag+P– group.

**Table 1:** Sample characteristics by neuropsychiatric symptom profile

Risk factors	Agitation and psychosis (Ag+P+) (n = 481)	Psychosis, but no agitation (Ag–P +) (n = 579)	Agitation, but no psychosis (Ag+P–) (n = 1325)	Neither agitation nor psychosis (Ag–P–) (n = 7721)	*P* value^a^
*Socio*-*demographic status and cognitive function*^*b*^					
Mean age at dementia diagnosis (SD)	81.3 (10.1)	81.8 (9.1)	81.0 (9.2)	81.1 (8.5)	0.267
Female gender (%)	62.6%^#^	69.3%*	57.6%*^,#^	63.7%^#^	< 0.001
Non-White ethnicity (%)	27.8%	31.3%*	23.3%^#^	24.0%^#^	< 0.001
Married or cohabiting status (%)	31.4%^#^	24.8%*	34.8%^#^	34.9%^#^	< 0.001
Mean index of deprivation (SD)	28.9 (10.3)*	28.2 (10.9)*	28.8 (10.7)*	26.6 (11.1)^#^	< 0.001
Mean MMSE score at diagnosis (SD)	15.6 (7.1)*^,#^	17.6 (6.5)*	15.3 (7.3)*^,#^	19.5 (6.0)^#^	< 0.001
Dementia subtype					< 0.001
Alzheimer’s disease	31.0%*	35.1%*	32.2%*	45.9%^#^	
Mixed-type dementia (including Alzheimer’s disease and Vascular dementia)	23.7%	24.5%	22.9%*	25.8%	
Vascular dementia	25.8%*^,#^	22.5%*	25.4%*	17.1%^#^	
Unspecified or other dementia	19.5%*	18.0%*	19.6%*	11.2%^#^	
*HoNOS symptoms/disorders (%)*^*b*^					
Non-accidental self-injury	4.4%*^,#^	1.4%	3.0%*^,#^	0.7%	< 0.001
Problem-drinking or drug taking	6.5%*^,#^	2.9%	5.4%*^,#^	2.5%	< 0.001
Depressed mood	27.0%*^,#^	20.9%*	21.9%*	12.5%^#^	< 0.001
Physical illness or disability	68.4%*^,#^	60.8%*	65.4%*	47.3%^#^	< 0.001
*HoNOS functional problems (%)*^*b*^					
Activities of daily living	82.5%*^,#^	70.6%*	81.3%*^,#^	51.6%^#^	< 0.001
Living conditions	21.3%*	18.9%*	18.0%*	9.3%^#^	< 0.001
Occupational/recreational activities	53.5%*^,#^	38.6%*	49.9%*^,#^	27.7%^#^	< 0.001
Social relationships	52.2%*^,#^	17.4%*	45.6%*^,#^	8.3%^#^	< 0.001
Antipsychotic prescription^c^	42.8%*^,#^	29.9%*	22.5%*^,#^	5.7%^#^	< 0.001
Hospitalisation prior to dementia diagnosis^d^	52.6%*	48.0%	50.8%*	46.1%	< 0.001

*Significantly different to group Ag–P– (*p* < 0.05); ^#^significantly different to group Ag–P + (*p* < 0.05)

^a^ANOVA or Chi^2^ test; ^b^at the time of dementia diagnosis; ^c^in a one-year window around dementia diagnosis; ^d^in the year prior to dementia diagnosis

### Hazards related to antipsychotic prescribing in the four neuropsychiatric symptom subgroups

Multivariate Cox regression models (Model 3; see Table 2) showed a 116% and significantly increased stroke hospitalisation risk associated with antipsychotic prescribing in the Ag–P + group, whereby no increased hazard was detected in any of the other groups.

**Table 2:** Risks of adverse outcomes in association with antipsychotic prescribing according to symptom group using Cox proportionate hazard models (Hazard ratios (95% CI))

	Mortality			Any emergency hospitalisation			Stroke hospitalisation			Stroke-specific mortality		
	Model 1	Model 2	Model 3	Model 1	Model 2	Model 3	Model 1	Model 2	Model 3	Model 1	Model 2	Model 3
Agitation and Psychosis (Ag+P+) (n = 481)	1.02 (0.81–1.28)	0.93 (0.74–1.18)	1.00 (0.78–1.27)	0.93 (0.74–1.16)	0.89 (0.71–1.11)	0.89 (0.71–1.13)	0.88 (0.40–1.94)	0.92 (0.41–2.07)	0.97 (0.42–2.21)	0.94 (0.46–2.04)	0.82 (0.40–1.71)	0.73 (0.33–1.61)
Psychosis, but no agitation (Ag–P +) (n = 579)	1.19 (0.95–1.49)	1.19 (0.95–1.49)	*1.26* *(1.00*–*1.60)*	*1.19* *(0.97*–*1.46)*	1.13 (0.92–1.39)	*1.20* *(0.97*–*1.48)*	*1.82* *(0.95*–*3.45)*	*1.86* *(0.97*–*3.57)*	**2.16** **(1.09–4.25)**	1.56 (0.84–2.91)	1.45 (0.77–2.73)	1.61 (0.82–3.13)
Agitation, but no psychosis (Ag+P–) (n = 1325)	**1.25** (**1.07**–**1.46**)	**1.18** (**1.00**–**1.38**)	*1.16* *(0.99*–*1.36)*	1.02 (0.87–1.20)	1.01 (0.86–1.19)	0.95 (0.80–1.11)	1.10 (0.65–1.87)	1.14 (0.67–1.95)	1.10 (0.64–1.88)	*1.53* *(0.97*–*2.41)*	*1.50* *(0.95*–*2.38)*	*1.53* *(0.97*–*2.43)*
Neither agitation nor psychosis (Ag–P–) (n = 7721)	**1.37** (**1.21**–**1.55**)	**1.17** (**1.03**–**1.33**)	*1.13* *(1.00*–*1.28)*	**1.14** **(1.02–1.28)**	1.03 (0.92–1.16)	0.97 (0.86–1.09)	1.10 (0.76–1.58)	0.99 (0.69–1.43)	0.97 (0.67–1.40)	**1.48** **(1.04–2.10)**	1.22 (0.86–1.74)	1.20 (0.84–1.71)

Model 1: Adjusted for age and gender

Model 2: Adjusted for age, gender, marital status, ethnicity, index of deprivation, MMSE score, and dementia subtype

Model 3: Adjusted for the above, HoNOS scores (non-accidental self-injury, problem-drinking or drug taking, depressed mood, physical illness or disability, activities of daily living, living conditions, occupational/recreational activities, social relationships), and hospitalisation in the year prior to dementia diagnosis

Bold *p* < 0.05

Italics 0.05 < *p* < 0.10

An interaction between antipsychotic prescribing and the Ag–P + group (when comparing to the hazard in all other subgroups combined) in relation to stroke hospitalisation could further be identified (*p* = 0.064) strengthening the finding that antipsychotic-related risk of hospitalisation due to stroke might be higher in this group (see Supplementary Table 2).

Whereby an increased antipsychotic mortality risk was detected in a Cox regression model adjusted for age, gender, marital status, ethnicity, index of deprivation, MMSE score, and dementia subtype (Model 2) in the Ag+P– and the Ag–P– group, this was no longer significant after further adjustment (Model 3). No increased risks of emergency hospitalisation or stroke-specific mortality were detected in relation to any of the groups in adjusted models (Model 2 and 3).

This was mirrored in non-significant interaction terms for the association between strata and antipsychotic prescribing in relation to the adverse health outcomes (see Supplementary Table 2). Although a 20% increased risk of emergency hospitalisation in the Ag–P + group only amounted to a non-significant trend (*p* = 0.096), the interaction term was significant (*p* = 0.042) indicating that antipsychotic-related emergency hospitalisation risk might be higher in patients with this symptom profile than in the remainder of the sample. The absence of evidence for an increased antipsychotic-related all-cause mortality risk by neuropsychiatric symptom strata was reflected the *p* values of the interaction terms. A significantly increased antipsychotic-related all-cause mortality risk was identified for those not in the A+P+ group. Together with *p* value of 0.076 for the interaction term this indicates that there might be a lower antipsychotic-related all-cause mortality risk in patients who don’t suffer from co-morbid agitation and psychosis.

### Hazards related to antipsychotic prescribing in the four dementia subtype groups

Multivariable Cox proportionate hazard models adjusted for age, gender, ethnicity, deprivation and MMSE (Model 2; see Table 3) showed an increased antipsychotic-related all-cause mortality and hospitalisation risk in patients diagnosed with Alzheimer’s disease, as well as an increased antipsychotic-related all-cause mortality risk in patients diagnosed with vascular dementia. After further adjustments for previous hospitalisation and HoNOS scores (including agitation and psychosis) a significantly increased antipsychotic-related mortality risk remained in patients diagnosed with Alzheimer’s disease (22% increase) and vascular dementia (29% increase). Further, patients with mixed-type dementia had a 65% and significantly increased risk of stroke hospitalisation associated with antipsychotic prescribing.

**Table 3:** Risks of adverse outcomes in association with antipsychotic prescribing according to dementia subtype diagnosis using Cox proportionate hazard models (Hazard ratios (95% CI))

	Mortality			Any emergency hospitalisation			Stroke hospitalisation			Stroke-specific mortality		
	Model 1	Model 2	Model 3	Model 1	Model 2	Model 3	Model 1	Model 2	Model 3	Model 1	Model 2	Model 3
Alzheimer’s disease (n = 4320)	**1.54** (**1.34–1.77**)	**1.34** (**1.16–1.54**)	**1.22** (**1.05–1.42**)	**1.22** (**1.07–1.39**)	**1.15** (**1.01–1.31**)	1.04 (0.91–1.20)	0.90 (0.53–1.53)	0.84 (0.49–1.44)	0.91 (0.52–1.58)	1.11 (0.64–1.92)	1.05 (0.60–1.83)	1.03 (0.57–1.83)
Vascular dementia (n = 1910)	**1.44** (**1.25–1.66**)	**1.38** (**1.19–1.60**)	**1.29** (**1.11–1.51**)	1.08 (0.93–1.25)	1.05 (0.91–1.22)	0.97 (0.83–1.13)	0.84 (0.54–1.30)	0.82 (0.53–1.28)	0.93 (0.59–1.48)	**1.39** **(1.00–1.93)**	*1.32* *(0.95*–*1.84)*	1.34 (0.94–1.91)
Mixed-type dementia (n = 2553)	1.04 (0.87–1.25)	0.94 (0.78–1.13)	0.90 (0.74–1.09)	1.03 (0.87–1.20)	0.97 (0.83–1.15)	0.96 (0.81–1.14)	*1.46* *(0.97*–*2.20)*	*1.44* *(0.95*–*2.19)*	**1.65** **(1.07–2.56)**	*1.53* *(0.94*–*2.48)*	1.48 (0.90–2.43)	1.42 (0.85–2.39)
Unspecified or other dementia (n = 1323)	*1.19* *(1.00*–*1.43)*	1.15 (0.97–1.38)	1.09 (0.90–1.32)	1.06 (0.89–1.27)	1.04 (0.87–1.24)	0.92 (0.76–1.12)	0.97 (0.53–1.78)	1.00 (0.55–1.84)	0.77 (0.40–1.50)	1.19 (0.65–2.18)	1.20 (0.65–2.20)	1.22 (0.64–2.34)

Model 1: Adjusted for age and gender

Model 2: Adjusted for age, gender, marital status, ethnicity, index of deprivation, and MMSE score

Model 3: Adjusted for the above, HoNOS scores (agitation, psychosis, non-accidental self-injury, problem-drinking or drug taking, depressed mood, physical illness or disability, activities of daily living, living conditions, occupational/recreational activities, social relationships), and hospitalisation in the year prior to dementia diagnosis

Bold *p* < 0.05

Italics 0.05 < *p* < 0.10

When testing for interactions (Supplementary Table 3), the increased antipsychotic related all-cause mortality risk was strengthened in the vascular dementia group (*p* = 0.090), but not in the Alzheimer’s disease group (*p* = 0.571). A significantly increased antipsychotic-related all-cause mortality risk was identified for those not in the mixed-type dementia group. Together with *p* value of 0.001 for the interaction term this indicates that there might be a lower antipsychotic-related all-cause mortality risk in patients with mixed-type dementia. For none of the other analyses *p* values for interaction < 0.1 were identified, but the there was an indication (*p* = 0.130) that the increased antipsychotic-related stroke risk in those diagnosed with mixed-type dementia might be true.

### Comparison of specific antipsychotics in relation to adverse outcomes

Cox proportionate hazard models evaluating specific antipsychotic medications against no antipsychotic or any other antipsychotic are presented in Table 4 (only Model 3) and Supplementary Table 1 (Models 1, 2, 3). Whilst, as aforementioned, an increased all-cause and stroke-specific mortality risk was identified for use of any antipsychotic compared to non-use, this could only be replicated with significant findings for second generation antipsychotics (15% increased all-cause mortality risk and 31% increased stroke-specific mortality risk compared to no use of an antipsychotic). Largest all-cause mortality risk increase appeared to be associated with risperidone, although this amounted to non-significant trend (*p* = 0.062). Direct comparisons in subgroups of antipsychotic users didn’t detect any significant differences between users of specific antipsychotics and users of any other antipsychotic; neither did the comparison between first- and second-generation antipsychotics.

**Table 4:** Risks of adverse outcomes according to specific antipsychotic type using Cox proportional hazard models (Hazard ratios (95% CI))

	Mortality	Any emergency hospitalisation	Stroke hospitalisation	Stroke-specific mortality
Any antipsychotic (n = 1115) versus no antipsychotic (n = 8991)	**1.14** **(1.04–1.24)**	0.99 (0.92–1.08)	1.09 (0.84–1.40)	**1.28** **(1.01–1.63)**
Risperidone (n = 283) versus any other antipsychotic (n = 832)	1.03 (0.86–1.22)	1.00 (0.84–1.18)	0.82 (0.46–1.44)	1.07 (0.65–1.75)
Risperidone (n = 283) versus no antipsychotic (n = 8991)	*1.16* *(0.99*–*1.36)*	0.99 (0.85–1.15)	0.93 (0.55–1.57)	1.34 (0.85–2.11)
Olanzapine (n = 162) versus any other antipsychotic (n = 953)	1.02 (0.81–1.29)	0.81 (0.65–1.00)	0.77 (0.36–1.61)	1.27 (0.68–2.35)
Olanzapine (n = 162) versus no antipsychotic (8991)	1.16 (0.93–1.45)	*0.83* *(0.67*–*1.01)*	0.86 (0.42–1.75)	1.58 (0.88–2.83)
Quetiapine (n = 402) versus any other antipsychotic (n = 713)	0.95 (0.82–1.10)	1.12 (0.97–1.30)	1.38 (0.88–2.16)	0.92 (0.60–1.41)
Quetiapine (n = 402) versus no antipsychotic (n = 8991)	1.11 (0.98–1.25)	1.07 (0.95–1.20)	1.30 (0.92–1.84)	1.22 (0.86–1.73)
Any second-generation (n = 889) antipsychotic versus no antipsychotic (n = 8991)	**1.15** **(1.05–1.26)**	1.02 (0.93–1.11)	1.13 (0.86–1.49)	**1.31** **(1.00–1.70)**
Any first-generation (n = 386) antipsychotic versus no antipsychotic (n = 8991)	1.10 (0.96–1.25)	0.94 (0.82–1.06)	1.06 (0.71–1.58)	1.12 (0.76–1.65)
Any first-generation antipsychotic (n = 223) versus any second-generation (n = 762) antipsychotic*	0.95 (0.79–1.14)	0.89 (0.74–1.07)	0.86 (0.48–1.57)	0.84 (0.49–1.44)

Adjusted for age, gender, marital status, ethnicity, index of deprivation, MMSE score, dementia subtype, HoNOS scores (agitation, psychosis, non-accidental self-injury, problem-drinking or drug taking, depressed mood, physical illness or disability, activities of daily living, living conditions, occupational/recreational activities, social relationships), and hospitalisation in the year prior to dementia diagnosis (Model 3)

Bold *p* < 0.05

Italics 0.05 < *p* < 0.10

The antipsychotic first mentioned can also be in combination (e.g. if risperidone prescribed in combination, it is grouped in the risperidone group)

* SGA versus FGA excludes combinations of second-generation with first-generation antipsychotics (n = 163)

## Discussion

In a large naturalistic sample of patients diagnosed in a specialist service for dementia and mental health care, we found that patients with dementia rated by clinicians to have problematic psychosis, but not co-morbid agitation or agitation alone, were at an increased risk of stroke associated with antipsychotic use.

A higher all-cause and stroke-specific mortality risk related to antipsychotic prescribing was detected in the whole sample. There was no true interaction between neuropsychiatric symptom strata and antipsychotic-related mortality risk, with the exception of a possibly higher antipsychotic-related mortality risk in those not in the agitation and psychosis group.

Further, an increased antipsychotic-related all-cause mortality risk was identified for patients with Alzheimer’s disease and vascular dementia, whereby an interaction between dementia subtype strata and antipsychotic mortality risk could only be identified for vascular dementia.

In the whole sample, second-generation antipsychotics were associated with an increased all-cause and stroke-related mortality risk compared to non-use, and this association amounted to a trend for risperidone. No other specific antipsychotic was significantly associated with a higher risk of adverse outcomes, neither in comparison to all other antipsychotic users nor non-users.

Almost half of those presenting with agitation and psychosis (Ag+P+) were prescribed antipsychotics, indicating a group in whom clinicians saw the greatest need for treatment, followed by those with psychosis alone, 30% of whom had antipsychotic treatment recorded. In our sample 24% of patients presented with neuropsychiatric symptoms at the time of dementia diagnosis, a symptom prevalence which is slightly lower than reported in previous research [3, 39]. The likely reason for this is that we excluded symptoms classified as ‘minor problem, requiring no action’ on the HoNOS scale, which might be included if a structured research scale is applied and recording is likely to be less rigorous in routinely collected data than in screened research samples.

The excess stroke risk associated with antipsychotic use in the Ag–P + group highlights challenges in the treatment of psychosis in dementia for which antipsychotics are frequently used. They are known to have at best modest efficacy in these circumstances and important side effect risks. In addition to the effects found in this study, antipsychotics are also associated with accelerated cognitive decline, sedation and extrapyramidal symptoms [6, 9, 40].

The results of this study differ from a meta-analysis data of data from 1721 patients included in randomised controlled trials of risperidone [12], which found that in those with delusions at baseline (as identified on the Behavioral Pathology in Alzheimer’s Disease (BEHAVE-AD) scale [41]), the risperidone-related stroke risk was not significantly increased (HR 1.47; 95% CI 0.59–3.65) as distinct from a raised risk in those who did not present with delusions (HR 5.88; 95% CI 2.09–16.53). Several explanations exist for these differences: While risperidone trials used a structured measure (BEHAVE-AD [41]), our classification of psychotic symptoms was according to the relevant HoNOS subscale. In this measure, the clinician rates if the neuropsychiatric symptom cluster poses a problem for the patient and their environment rather than describing the nature of the psychotic symptom. Further the inclusion criteria differed between the meta-analysis and our study. Risperidone trials [12] only included patients if they displayed psychosis or scored above a threshold on BEHAVE-AD [41], leading to a mixed population of patients with psychosis, agitation or a combination of neuropsychiatric symptoms. Our study aimed to examine agitation and psychosis as combined or mutually exclusive. We aimed to assess a class effect of antipsychotics in relation to neuropsychiatric symptom profiles and did not investigate specific properties of individual medications in relation to the strata. It should also be borne in mind that clinical trials inevitably recruit rather selected samples, whereas our cohort may be more representative of the treated population.

There are several explanations why patients with dementia presenting with psychotic symptoms might be at a higher risk of antipsychotic-related stroke. First, the presence of tau pathology has both been linked to the presence of psychotic symptoms and an adverse prognosis in Alzheimer’s disease [17, 42, 43]. Evidence from mouse models suggests that tau mediates excitotoxicity after cerebrovascular events [44]. It is therefore possible that small cerebrovascular events triggered by antipsychotic-related excessive sedation, dehydration or orthostatic hypotension [45] might lead to a deficit in brain perfusion, and the response to this deficit might be exaggerated to toxic levels in the presence of tau pathology, ultimately leading to a hospitalised stroke [44]. In addition, a recent study of autopsied cases of AD demonstrated that cerebral amyloid angiopathy and advanced small vessel disease were more common in AD patients with psychosis than in those without psychosis [15]. Although its role in the neurodegenerative process is not fully understood, cerebral amyloid angiopathy weakens cerebral blood vessels [46] and can lead to microbleeds or larger haemorrhages. It is therefore possible that psychosis in Alzheimer’s disease is related to vascular fragility, and antipsychotics might increase risk of stroke via the aforementioned mechanisms.

Moreover, there is an increasing recognition that patients diagnosed with a single dementia subtype, as Alzheimer’s disease, frequently have co-morbid cerebral pathologies, as Lewy bodies, vascular pathology, or TDP-43 proteinopathy, when autopsied [47]. The presence of Lewy bodies is associated with a higher frequency of visual hallucinations [47–49] as well as with sensitivity reactions to antipsychotic medications, and might thereby explain in part the increased risk of adverse outcomes [16, 50].

We didn’t not find evidence for an increased antipsychotic-related stroke risk in patients with agitation, both in those with and those without co-morbid psychosis. While pathological and anatomical changes in psychosis in dementia have been extensively studied and also distinguished from psychosis in schizophrenia [51], less is known about agitation in dementia. Agitation in dementia has been associated with frontal lobe dysfunction [52] and brain regions involved in subjective emotional experiences [53]. It has been hypothesised that agitation could arise from overestimating or misinterpreting potential threats [54], whereby those threats could for example be pain or changes in the environment. It is however also conceivable that, as possibly in the agitation and psychosis group, those overinterpreted threats are psychotic or quasi psychotic experiences. Further research is required to clarify the neuropathological corelates of agitation in dementia, especially distinguishing between ‘agitation as a syndrome’ from ‘agitation as response to another disorder’ (e.g. agitation and psychosis) [52], which could also elucidate possible mechanisms for differences in antipsychotic hazards compared to those with only psychosis.

When we stratified by dementia subtype, we identified a robust 29% increased antipsychotic related mortality risk in patients diagnosed with vascular dementia. This is different from a previous study by Sultana and colleagues in this data source [30], which might be grounded in two key differences between the two studies: First, we ascertained prevalent use of antipsychotic medication at dementia diagnosis and the previous study antipsychotic use at any timepoint after dementia diagnosis. Second, Sultana and colleagues only included second-generation (atypical) antipsychotics, while our study evaluated the impact of all classes of antipsychotics and first-generation antipsychotics have recently been shown to yield a higher mortality risk in those with cardiovascular or cerebrovascular disease [55].

We could only identify a class effect for all antipsychotics and second-generation antipsychotics in relation to mortality risk. Although no significant results were obtained, there was a trend towards a higher mortality risk in those taking risperidone, which was also the largest group taking a specific antipsychotic. In line with previous research [56], Quetiapine appeared to yield lowest risk of mortality. The absence of evidence for increased hazards in the first-generation antipsychotic group is in line with a recent study [57] showing that patients with Alzheimer’s disease using first-generation antipsychotics had a lower risk of death than those using second-generation antipsychotics.

Finally, although this cannot be derived from our data, the dose and antipsychotic agent used might differ between prescription for agitation and for psychosis in dementia. While clear guidance exists in the UK and Europe for cautious management of agitation [10, 58], prescribing for psychosis is off-label and clinicians might use higher doses leading to an increased excess risk of harm [56].

Strengths of this study include the large naturalistic sample of patients diagnosed with dementia by a near-monopoly dementia care provider for its catchment population. The use of clinician-rated real-world measures of neuropsychiatric problems should also give a clinically relevant picture. MMSE scores in our sample were higher than in most clinical trials conducted on antipsychotics in people with dementia, which largely include participants in the severe stage of illness [12]. For this reason, similar to CATIE-AD [59], our findings might be more applicable in earlier disease stages and thereby more relevant to patients and their families. The richness of this data allowed adjustment for a wide range of confounders, and the linkage to national data on mortality and hospitalisation should have resulted in close to complete ascertainment of outcome data. A particular advantage of NLP is that the applications account for the linguistic context of a statement of interest in free text, enabling exclusion of negation and other irrelevant statements and ascertaining current, rather than past, prescription or speculations about future prescribing [29]. However, the use of routinely collected electronic health record data also presents a number of limitations. First, consistent recording of patient information is only available at certain time points of the patient’s journey through the healthcare system. Therefore, we chose to ascertain recording of neuropsychiatric symptoms and antipsychotic prescription in the most reliable window around the time of first dementia diagnosis. Second, we could extract information on the initiation of antipsychotic agents and on use at given times, but we were not able to track antipsychotic prescription longitudinally. However, hazardous effects of antipsychotics are already present when only prescribed short-term [60] and the agents are unlikely to be withdrawn in the absence of effective alternative interventions [61]. Nevertheless, exclusively considering exposure to antipsychotics around the time of diagnosis leads to individuals prescribed antipsychotics in a later stage of dementia not being considered as exposed, which could potentially bias estimates towards the null and is particularly relevant to the null finding in the relatively small Ag+P+ group. This group is more than 2.5 times smaller than the other groups, which might have resulted in a lack of power in adjusted analyses. Third, in addition to the aforementioned challenges with data availability and temporality further limitations of ascertaining variables through NLP from clinical records need to be acknowledged: The output depends on the accuracy and quality of data entry, which varies by individual clinician and is compromised through the use of jargon, idiosyncratic abbreviations or misspellings [21]. Although precision and recall are relatively high for the medication NLP application, there remains a risk under- or overestimating the true prescribing prevalence. Moreover, there is a possibility of misclassification of the reason for stroke-related hospitalisation or death. As we only used the first discharge diagnosis in order to ascertain new cerebrovascular events, strokes that occurred in the context of another medical event might have been missed, potentially leading to under-recording and underestimation of effects. The broader definition of the stroke outcome according to ICD-10 was used to reflect the uncertainty which often exists around cerebrovascular events in hospital settings, where distinction from TIA or other ‘stroke mimics’ is often not possible in the short time-frame of the admission [33], but this approach might also lead to a higher detection of false positives. That we are only detecting an increased antipsychotic-related risk of hospitalised stroke in the Ag–P +, but no increased risk of stroke-related mortality, could reflect that those patients are experience signs of stroke but not necessarily cerebrovascular changes. Conversely, death certificate data is prone to classification bias and sensitivity for recording of stroke on death certificate has been shown to be below 71% in most studies [62]. Fourth, we were only able to examine individual antipsychotics across the whole sample and did not seek to analyse by type of antipsychotic or individual agent in analyses stratified by neuropsychiatric symptom profile or subtype diagnosis. Heterogeneity by agent or sub-class can therefore not be excluded in these analyses. Fifth, physical health was ascertained through previous hospitalisation and the HoNOS ‘Physical illness and disability problems’ subscale. The latter, although widely used in clinical mental health services in the UK and consistently showing relations with important outcomes [26], is a relatively brief measure without detailed information on the conditions determining its score. Sixth, data on level of education was not available, and socio-economic status was only ascertained at neighbourhood rather than individual level [34]. Thus, although we were able to adjust for a range of demographic, clinical, social and functional covariates, residual confounding cannot be fully excluded. This is particularly relevant for smaller subgroups (as Ag+P+) where adjustment for confounders might not fully account for differences in baseline variables and correlation does not necessarily infer causality. An important consideration for pharmacoepidemiologic studies is confounding by indication, whereby individuals with more severe symptoms are more likely to be prescribed medications. We stratified patients by the presence of agitation and/or psychosis according to a well-being score but could not measure severity of these symptoms. As stroke prior to onset of Alzheimer’s disease has been described as associated with a higher risk of delusions [14], higher levels of cerebrovascular morbidity might be present a priori in patients with psychosis prescribed an antipsychotic. Lastly, for the purpose of this study we only examined whether antipsychotic-related hazards differed in relation to agitation and psychosis as these are the two main indications for antipsychotic use in dementia. Future studies could examine whether degree of cognitive impairment, overall neuropsychiatric symptom burden or other specific neuropsychiatric symptoms (e.g. depression or anxiety) affect antipsychotic hazards, which could feed into more advanced prediction models and potentially translate into real-time risk detection and alerting systems [63].

## Conclusions

Our data suggests that antipsychotic use is associated with more than a doubling of risk of cerebrovascular events in patients with dementia suffering from psychosis (without agitation). Advocacy for avoidance of antipsychotics has been strongest in for agitation in dementia [58], but this might be equally, or even more important, in those presenting with psychosis. While non-pharmacological management strategies of psychosis are becoming more accepted in the field of functional psychotic disorders [64], there is little evidence available on the efficacy of such interventions in dementia [65] and novel strategies to address distressing psychotic symptoms in dementia clearly need further development and evaluation.

## Electronic supplementary material

Below is the link to the electronic supplementary material.

Supplementary material 1 (DOCX 43 kb)

Supplementary material 2 (DOCX 27 kb)
