# Supplementary material for: Antipsychotic use in dementia: the relationship between neuropsychiatric symptom profiles and adverse outcomes
Source: Eur J Epidemiol. 2020 May 15;36(1):89–101. doi: 10.1007/s10654-020-00643-2 (PMC7847435; doi:10.1007/s10654-020-00643-2)
Supplement: Supplementary file 1 — Supplementary material 1 (DOCX 43 kb) [file 10654_2020_643_MOESM1_ESM.docx]

**Antipsychotic use in dementia: The relationship between neuropsychiatric symptom profiles and adverse outcomes**

**Supplementary document: Ascertainment of variables from structured fields and through natural language processing on the CRIS resource**

1. Summary of generic procedures for natural language processing (NLP) application development in CRIS

NLP applications are developed using the GATE (General Architecture for Text Engineering; [www.gate.ac.uk](http://www.gate.ac.uk)) software (1, 2). This is a development environment for writing applications that can process human language. Its principal purpose is to extract required information as structured data from free text fields using algorithms developed for this purpose. GATE was originally developed at the University of Sheffield beginning in 1995, and a longstanding collaboration between the BRC Nucleus, which hosts the CRIS system, and engineers at University of Sheffield supports the development of new apps, as well as their maintenance and staff training.

Each application is developed through expert annotation, whereby domain experts code whether the variable of interest is present in the current document having agreed pre-defined coding rules. In general, the interest is in ascertaining positive mentions of given entities being present. Negation statements (e.g., relevant to this manuscript, ‘not using any antipsychotic’) are therefore not specifically captured as entities (3), but are combined with other unwanted text mentions in classification and performance estimation.

NLP application performance metrics are focused on precision and recall. Precision (positive predictive value) is the proportion of algorithm-derived named entities that are judged to be correct. Recall (sensitivity) is the proportion of gold standard named entities that are identified by the algorithm. Performance is evaluated by running the NLP application over a corpus of unseen documents, identifying and examining the original clinical document through the linked document ID, and comparing the results to the manual and NLP coding (3).

1. Table: Ascertainment of baseline and co-variates from the CRIS resource

| **Variable** | **Source** |
| --- | --- |
| **Socio-demographic status and cognitive function** | |
| Age at dementia diagnosis | Date of birth pseudonymised to 01/month/year from structured field in source record.  First date of dementia diagnosis as per ‘Diagnosis’ NLP application^1^. |
| Gender | Structured field |
| Ethnicity | Structured field |
| Married or cohabiting status | Structured field |
| Index of deprivation | Structured address field identifies the Lower Super Output Area which is linked to an index of deprivation derived from the 2011 Census |
| MMSE | ‘MMSE’ NLP application^2^ supplemented by structured field recording of the total numerator and denominator score. |
| **Dementia subtype** | |
| Alzheimer’s disease | According to ICD-10 (F00.1) from structured field (where clinician enters the ICD-10 code and the ‘diagnosis’ NLP application^1^ (additionally allowing text strings with the diagnostic statement Alzheimer*) |
| Mixed-type dementia (including Alzheimer’s disease and vascular dementia) | According to ICD-10 (F00.2) from structured field (where clinician enters the ICD-10 code and the ‘diagnosis’ NLP application^1^ (additionally allowing text strings with the diagnostic statement ‘Alzheimer*’); mixed-type dementia ascertained when both vascular dementia and Alzheimer’s disease present in the patients record. |
| Vascular dementia | According to ICD-10 (F01) from structured field (where clinician enters the ICD-10 code) and the ‘diagnosis’ NLP application^1^ (additionally allowing text strings with the diagnostic statement ‘vascular dementia’) |
| Unspecified or other dementia | According to ICD-10 (F03) from structured field (where clinician enters the ICD-10 code) or in the absence of a specific diagnostic code or statement (including the term ‘dementia’ identified in NLP). |
| **HoNOS65+ symptoms/disorders** | |
| Non-accidental self-injury | Structured field |
| Problem-drinking or drug taking | Structured field |
| Depressed mood | Structured field |
| Physical illness or disability | Structured field |
| **HoNOS65+ functional problems** | |
| Activities of daily living | Structured field |
| Living conditions | Structured field |
| Occupational / recreational activities | Structured field |
| Social relationships | Structured field |
| **Antipsychotic prescription** | ‘Medication’ NLP application^3^ supplemented by structured fields in the source record (dedicated medication form, treatment plan, mental health trust pharmacy dispensary). |

^1,2,3^Descriptions of the ‘Medication’ (3.), ‘Diagnosis’ (4.) and ‘MMSE’ (5.) applications follow below.

3. Medication NLP application:

**3.1 Description:**

The medications application is designed to extract the names of medications that can be inferred as currently prescribed to the patient (4, 5). Groups or classes of medications are assigned through post-processing of derived data. For the analyses described in the accompanying manuscript, the application was used to identify antipsychotics prescribed within a window of 6 months either side of first dementia diagnosis, and it set to not capture medication prescription assumed to be in the past and medications which might hypothetically be prescribed in the future (for example, statements in text indicating that a patient should be prescribed a certain drug if their condition worsens but not indicative of a current prescription).

**3.2 Pipeline:**

The GATE software codes all elements of a text document. Using a gazetteer it labels all instances of medications from the British National Formulary, including a locally generated list of common misspellings of psychotropic agents. Further gazetteers are applied to identify time and route of administration as well as dose frequency. Bespoke rules are applied to ascertain whether a given medication identified represents a statement implying current use. The app preferentially detects current use of medications (a) where there is corresponding dosage information, (b) where present use is explicitly mentioned (Recommendations to GPs, ‘Current medication:’, ‘Plan:’, ‘Prescribed’, ‘Currently on’), or (c) where this is discernible through inference (e.g. ‘patient’ is faring better on risperidone’).

Using information from the NLP application, use is ascertained of specific medications or medications within a particular category (e.g. antipsychotics) as part of a bespoke data extraction for a project in question. This is supplemented by structured field data from a dedicated medication form or treatment plan in the source record, and further information on certain specifically-dispensed agents is available in CRIS from the Trust’s pharmacy database. Structured fields of this sort are uncommonly used to record medication information in people with dementia and in a search on 8^th^ January 2020 77% of all medication recordings in people with dementia on CRIS were identified in free text.

**3.3 Illustrative examples of identification of risperidone in text:**

3.3.1 Positive examples (risperidone inferred as currently prescribed)

‘Plan Restart Risperidone 4mg from today and script written.’

‘Plan Continue Risperidone 2mg OD.’

‘Plan Increase risperidone by 0.5mg to 2.5mg’

‘At present (patient) is currently on Risperidone 4mg nocte’

‘Now on 8mg risperidone up from 6mg.’

‘(patient) was given a prescription for 56 days for Risperidone 2mg BD’

3.3.2 Negative examples (text classified as insufficient for inferring a current prescription of risperidone)

‘We will review whether (patient) wants to continue this or switch to an alternative depot such as low dose depixol or try oral risperidone when she is ready.’

‘History of poor compliance with medications, has been on risperidone, clozapine in the past.’

‘(Relative) concerned that Risperidone no longer effective in controlling symptoms.’

‘repeated problems with psychosis/manic episodes, treated with Risperidone and Valproate, subsequently diagnosed as CMV encephalitis.’

‘Referred by emergency department following complaining of taking an overdose of risperidone’

‘(Patient) was visited by her husband during the shift and received a leaflet about Risperidone.’

**3.4 Validation and performance metrics for antipsychotic prescription**

Precision was tested on prescription of any antipsychotic through manual checking 200 documents across the whole of CRIS which had been identified as containing a prescription of an antipsychotic by NLP software. The precision was 81%.

Precision was next explicitly tested for the antipsychotics olanzapine (in 20 documents), quetiapine (in 40 documents) and risperidone (in 20 documents). Precision for current use was 90% for olanzapine, 83% for quetiapine and 95% for risperidone.

Recall was tested for ever use of an antipsychotic on 52 documents and found to be 77%.

4. Diagnosis NLP application:

**4.1 Description:**

The application extracts text strings indicating a patient’s recorded diagnosis and the date the respective diagnosis was recorded. It is primarily intended to supplement diagnosis data from structured fields, which generally have high rates of completion but more limited updating following changes in diagnosis, or limited recording of comorbid diagnoses such as personality disorder.

**4.2 Pipeline:**

Search terms are derived from a gazetteer of diagnoses and ICD10 codes. The following coding rules were applied when developing the application and it identifies the following features from free text:

‘...Diagnosis: Fxx.x diagnosis name...’

‘...Diagnosis Fxx.x diagnosis name...’

‘...Diagnosis: diagnosis name...’

‘...Diagnosis: Fxx.x...’

Other features added were names of diagnoses for which no clear ICD-10 code exists (e.g. dementia with Lewy bodies (6)), potential misspellings of diagnoses, and exclusions of hypothetical and non-psychiatric diagnoses.

As aforementioned, the application is used to supplement structured field data, as there is dedicated field in the source record, which is mandatory to complete for clinicians. In a search conducted on 8^th^ January 2020 69% of data points indicating a diagnosis in CRIS were in structured fields. For the analysis described in the accompanying manuscript, the first diagnostic statement indicating a dementia diagnosis after cohort entry was used as a case definition, either via NLP or from a structured field depending on which recording occurred first.

**4.3 Validation and performance metrics:**

The diagnosis application has been validated for a number of diagnoses. For example, in relation to vascular dementia 75 documents were manually examined, which had been coded by GATE to determine this diagnosis. Precision was found to be 99% and recall 98% (5). In a validation for Alzheimer’s disease a precision of 98% and recall of 88% were observed when combining structured and free-text diagnoses and examining the full patient history.

5. MMSE (Mini Mental State Examination) NLP application:

**5.1 Description:**

The application extracts occurrences of MMSE scores and returns the MMSE score (subdivided into numerator and denominator) and the associated date.

**5.2 Pipeline:**

The application identifies MMSE scores associated with a date. The construct sought is ‘What MMSE score did the patient attain on a certain date?’. The application is supplemented by information from a structured field for MMSE score from the source record and in a search conducted on 8^th^ January 2020, 89% of all data points containing an MMSE recording were derived from free text. For the analysis described in the accompanying manuscript, the MMSE score closest to first dementia diagnosis was extracted.

**5.3 Validation and performance metrics:**

The MMSE application has been evaluated on 100 documents and yielded a precision of 97% and a recall of 98% for numerator and denominator (7). False positive instances were only seen when MMSE score had already been flagged in the document and it was raised twice, or another irrelevant score had been picked up. Occasionally, false positives occurred when the statement was questioning the MMSE score e.g. ‘/15, /20?’. Technically incorrect dates raised were most often within a matter of days of the correct date or occurred when there were multiple dates in the comment and it was unclear what date defined what event (date precision for same day was 68%, for within one week was 76%, for within two weeks 81%, and for within one month 84%; date recall was 88%).

References:

1. Cunningham H. GATE, a general architecture for text engineering. Comput Hum. 2002;36:223-54.

2. Cunningham H, Tablan V, Roberts A, Bontcheva K. Getting more out of biomedical documents with GATE's full lifecycle open source text analytics. PLoS computational biology. 2013;9(2):e1002854.

3. Perera G, Broadbent M, Callard F, et al. Cohort profile of the South London and Maudsley NHS Foundation Trust Biomedical Research Centre (SLaM BRC) Case Register: current status and recent enhancement of an Electronic Mental Health Record-derived data resource. BMJ Open. 2016;6(3):e008721. doi:10.1136/bmjopen-2015-008721

4. Kadra G, Stewart R, Shetty H, et al. Extracting antipsychotic polypharmacy data from electronic health records: developing and evaluating a novel process. BMC Psychiatry. 2015;15:166. doi:10.1186/s12888-015-0557-z

5. Sultana J, Chang CK, Hayes RD, et al. Associations between risk of mortality and atypical antipsychotic use in vascular dementia: a clinical cohort study. Int J Geriatr Psychiatry. 2014;29(12):1249-54. doi:10.1002/gps.4101

6. Mueller C, Perera G, Rajkumar A, et al. Hospitalization in people with dementia with Lewy bodies: Frequency, duration, and cost implications. Alzheimer's & Dementia: Diagnosis, Assessment & Disease Monitoring. 2018;10:143-52.

7. Su YP, Chang CK, Hayes RD, et al. Mini-mental state examination as a predictor of mortality among older people referred to secondary mental healthcare. PLoS One. 2014;9(9):e105312. doi:10.1371/journal.pone.0105312
