# Supplementary material for: Antipsychotic use in dementia: the relationship between neuropsychiatric symptom profiles and adverse outcomes
Source: Eur J Epidemiol. 2020 May 15;36(1):89–101. doi: 10.1007/s10654-020-00643-2 (PMC7847435; doi:10.1007/s10654-020-00643-2)
Supplement: Supplementary file 2 — Supplementary material 2 (DOCX 27 kb) [file 10654_2020_643_MOESM2_ESM.docx]

**Supplementary Tables:**

Supplementary Table 1: Risks for adverse outcomes according to specific antipsychotic type using Cox proportional hazard models (Hazard ratio (95% CI))

|  | Mortality | | |  | Any Emergency Hospitalisation | | |  | Stroke Hospitalisation | | |  | Stroke-specific Mortality | | |
| --- | --- | --- | --- | --- | --- | --- | --- | --- | --- | --- | --- | --- | --- | --- | --- |
|  | Model 1 | Model 2 | Model 3 |  | Model 1 | Model 2 | Model 3 |  | Model 1 | Model 2 | Model 3 |  | Model 1 | Model 2 | Model 3 |
| Any antipsychotic (n=1,115) vs. no antipsychotic (n=8,991) | **1.43**  **(1.32-1.54)** | **1.22 (1.13-1.32)** | **1.14 (1.05-1.24)** |  | **1.17 (1.09-1.26)** | 1.07 (0.99-1.15) | 0.99 (0.92-1.08) |  | 1.12 (0.88-1.41) | 1.02 (0.80-1.29) | 1.09 (0.84-1.40) |  | **1.54 (1.23-1.93)** | **1.27 (1.02-1.60)** | **1.28 (1.01-1.63)** |
| Risperidone (n=283) vs. any other antipsychotic (n=832) | 1.02 (0.86-1.20) | 1.08 (0.91-1.29) | 1.03 (0.86-1.22) |  | 0.99 (0.84-1.17) | 1.05 (0.89-1.24) | 1.00 (0.84-1.18) |  | 0.77 (0.44-1.35) | 0.79 (0.45-1.39) | 0.82 (0.46-1.44) |  | 0.97 (0.59-1.58) | 1.06 (0.65-1.73) | 1.07 (0.65-1.75) |
| Risperidone (n=283) vs. no antipsychotic (n=8,991) | **1.45 (1.24-1.68)** | **1.30 (1.11-1.51)** | *1.16 (0.99-1.36)* |  | **1.17 (1.01-1.35)** | 1.10 (0.95-1.28) | 0.99 (0.85-1.15) |  | 0.91 (0.54-1.51) | 0.84 (0.51-1.41) | 0.93 (0.55-1.57) |  | *1.51 (0.97-2.33)* | 1.33 (0.86-2.06) | 1.34 (0.85-2.11) |
| Olanzapine (n=162) vs. any other antipsychotic (n=953) | 1.09 (0.87-1.37) | 1.03 (0.82-1.30) | 1.02 (0.81-1.29) |  | 0.90 (0.72-1.11) | 0.84 (0.68-1.05) | 0.81 (0.65-1.00) |  | 0.80 (0.38-1.66) | 0.80 (0.38-1.68) | 0.77 (0.36-1.61) |  | 1.27 (0.69-2.33) | 1.27 (0.69-2.35) | 1.27 (0.68-2.35) |
| Olanzapine (n=162) vs. no antipsychotic (8,991) | **1.54 (1.25-1.92)** | **1.25 (1.00-1.56)** | 1.16 (0.93-1.45) |  | 1.07 (0.87-1.31) | 0.92 (0.75-1.13) | *0.83 (0.67-1.01)* |  | 0.91 (0.45-1.84) | 0.84 (0.41-1.70) | 0.86 (0.42-1.75) |  | **1.90 (1.07-3.37)** | 1.57 (0.88-2.81) | 1.58 (0.88-2.83) |
| Quetiapine (n=402) vs. any other antipsychotic (n=713) | 0.94 (0.82-1.09) | 0.90 (0.78-1.05) | 0.95 (0.82-1.10) |  | 1.13 (0.98-1.30) | 1.08 (0.94-1.25) | 1.12 (0.97-1.30) |  | 1.39 (0.89-2.17) | 1.35 (0.86-2.12) | 1.38 (0.88-2.16) |  | 0.93 (0.61-1.42) | 0.89 (0.58-1.35) | 0.92 (0.60-1.41) |
| Quetiapine (n=402) vs. no antipsychotic (n=8,991) | **1.38 (1.23-1.55)** | **1.15 (1.02-1.29)** | 1.11 (0.98-1.25) |  | **1.26 (1.13-1.41)** | *1.12 (1.00-1.25)* | 1.07 (0.95-1.20) |  | *1.34 (0.96-1.87)* | 1.20 (0.86-1.68) | 1.30 (0.92-1.84) |  | **1.48 (1.05-2.07)** | 1.18 (0.84-1.67) | 1.22 (0.86-1.73) |
| Any second-generation (n=889) antipsychotic vs. no antipsychotic (n=8,991) | **1.41 (1.29-1.53)** | **1.21 (1.11-1.33)** | **1.15 (1.05-1.26)** |  | **1.19 (1.10-1.29)** | *1.08 (1.00-1.18)* | 1.02 (0.93-1.11) |  | 1.13 (0.87-1.47) | 1.04 (0.80-1.35) | 1.13 (0.86-1.49) |  | **1.52 (1.18-1.94)** | *1.28 (1.00-1.65)* | **1.31 (1.00-1.70)** |
| Any first-generation (n=386) antipsychotic vs. no antipsychotic (n=8,991) | **1.48 (1.30-1.67)** | **1.22 (1.08-1.39)** | 1.10 (0.96-1.25) |  | **1.15 (1.01-1.29)** | 1.02 (0.91-1.16) | 0.94 (0.82-1.06) |  | 1.11 (0.75-1.65) | 1.01 (0.68-1.50) | 1.06 (0.71-1.58) |  | **1.46 (1.00-2.13)** | 1.16 (0.79-1.69) | 1.12 (0.76-1.65) |
| Any first-generation antipsychotic (n=223) vs. any second- generation (n=762) antipsychotic* | 1.08 (0.90-1.29) | 1.01 (0.84-1.22) | 0.95 (0.79-1.14) |  | 0.94 (0.78-1.12) | 0.92 (0.76-1.10) | 0.89 (0.74-1.07) |  | 0.94 (0.52-1.70) | 0.92 (0.51-1.67) | 0.86 (0.48-1.57) |  | 1.01 (0.59-1.70) | 0.91 (0.53-1.54) | 0.84 (0.49-1.44) |

**Model 1:** Adjusted for age and gender

**Model 2:** Adjusted for age, gender, marital status, ethnicity, index of deprivation, MMSE score, and dementia subtype

**Model 3:** Adjusted for the above, HoNOS scores (agitation, psychosis, non-accidental self-injury, problem-drinking or drug taking, depressed mood, physical illness or disability, activities of daily living, living conditions, occupational / recreational activities, social relationships), and hospitalisation in the year prior to dementia diagnosis

**Bold** p<0.05

***Italics*** 0.05<p<0.10

Antipsychotic first mentioned can also be in combination (e.g. if Risperidone prescribed in combination, in Risperidone group) other group might still combinations, but not Risperidone

* SGA vs. FGA excludes combinations of second generation with first generation antipsychotics (n=163)

Supplementary Table 2: Interaction between antipsychotic prescribing x symptom profile in relation to adverse health outcomes

|  | All-cause Mortality | | |  | Any Emergency Hospitalisation | | |  | Stroke Hospitalisation | | |  | Stroke-specific Mortality | | |
| --- | --- | --- | --- | --- | --- | --- | --- | --- | --- | --- | --- | --- | --- | --- | --- |
|  | Symptom present (HR, 95% CI) | Symptom not present  (HR, 95% CI) | P (Interaction) |  | Symptom present (HR, 95% CI) | Symptom not present  (HR, 95% CI) | P (Interaction) |  | Symptom present (HR, 95% CI) | Symptom not present  (HR, 95% CI) | P (Interaction) |  | Symptom present (HR, 95% CI) | Symptom not present  (HR, 95% CI) | P (Interaction) |
| Agitation & Psychosis (Ag+P+) | 1.00  (0.78-1.27) | **1.21**  **(1.11-1.32)** | *0.076* |  | 0.89  (0.71-1.13) | 1.02  (0.94-1.11) | 0.327 |  | 0.97  (0.42-2.21) | 1.07  (0.82-1.38) | 0.774 |  | 0.73  (0.33-1.61) | **1.34**  **(1.05-1.71)** | 0.288 |
| Psychosis, but no agitation (Ag-P+) | *1.26*  *(1.00-1.60)* | **1.13**  **(1.04-1.24)** | 0.443 |  | *1.20*  *(0.97-1.48)* | 0.96  (0.88-1.04) | **0.042** |  | **2.16**  **(1.09-4.25)** | 0.93  (0.71-1.22) | *0.064* |  | 1.61  (0.82-3.13) | 1.18  (0.92-1.52) | 0.481 |
| Agitation, but no psychosis (Ag+P-) | *1.16*  *(0.99-1.36)* | **1.14**  **(1.04-1.25)** | 0.994 |  | 0.95  (0.80-1.11) | 1.02  (0.93-1.11) | 0.392 |  | 1.10  (0.64-1.88) | 1.03  (0.78-1.35) | 0.813 |  | *1.53*  *(0.97-2.43)* | 1.16  (0.89-1.52) | 0.367 |
| Neither agitation nor psychosis (Ag-P-) | *1.13*  *(1.00-1.28)* | *1.10*  *(0.99-1.23)* | 0.495 |  | 0.97  (0.86-1.09) | 1.01  (0.90-1.12) | 0.774 |  | 0.97  (0.67-1.40) | 1.19  (0.83-1.70) | 0.437 |  | 1.20  (0.84-1.71) | 1.27  (0.92-1.77) | 0.910 |

Adjusted for age, gender, marital status, ethnicity, index of deprivation, MMSE score, dementia subtype, HoNOS scores (non-accidental self-injury, problem-drinking or drug taking, depressed mood, physical illness or disability, activities of daily living, living conditions, occupational / recreational activities, social relationships), and hospitalisation in the year prior to dementia diagnosis (Model 3)

**Bold** p<0.05

***Italics*** 0.05<p<0.10

Supplementary Table 3: Interaction between antipsychotic prescribing x subtype diagnosis in relation to adverse health outcomes

|  | All-cause Mortality | | |  | Any Emergency Hospitalisation | | |  | Stroke Hospitalisation | | |  | Stroke-specific Mortality | | |
| --- | --- | --- | --- | --- | --- | --- | --- | --- | --- | --- | --- | --- | --- | --- | --- |
|  | Diagnosis present (HR, 95% CI) | Diagnosis not present  (HR, 95% CI) | P (Interaction) |  | Diagnosis present (HR, 95% CI) | Diagnosis not present  (HR, 95% CI) | P (Interaction) |  | Diagnosis present (HR, 95% CI) | Diagnosis not present  (HR, 95% CI) | P (Interaction) |  | Diagnosis present (HR, 95% CI) | Diagnosis not present  (HR, 95% CI) | P (Interaction) |
| Alzheimer’s disease | **1.22**  **(1.05-1.42)** | **1.15**  **(1.04-1.28)** | 0.571 |  | 1.04  (0.91-1.20) | 0.98  (0.89-1.08) | 0.370 |  | 0.91  (0.52-1.58) | 1.15  (0.87-1.53) | 0.509 |  | 1.03  (0.57-1.83) | **1.41**  **(1.08-1.84)** | 0.194 |
| Vascular dementia | **1.29**  **(1.11-1.51)** | **1.11**  **(1.01-1.23)** | *0.090* |  | 0.97  (0.83-1.13) | 1.01  (0.92-1.11) | 0.706 |  | 0.93  (0.59-1.48) | 1.19  (0.88-1.61) | 0.250 |  | 1.34  (0.94-1.91) | 1.23  (0.89-1.71) | 0.614 |
| Mixed-type dementia | 0.90  (0.74-1.09) | **1.25**  **(1.13-1.37)** | **0.001** |  | 0.96  (0.81-1.14) | 1.01  (0.92-1.11) | 0.201 |  | **1.65**  **(1.07-2.56)** | 0.95  (0.69-1.29) | 0.130 |  | 1.42  (0.85-2.39) | 1.34  (1.02-1.76) | 0.867 |
| Unspecified or other dementia | 1.09  (0.90-1.32) | **1.17**  **(0.07-1.29)** | 0.435 |  | 0.92  (0.76-1.12) | 1.02  (0.93-1.11) | 0.762 |  | 0.77  (0.40-1.50) | 1.18  (0.90-1.55) | 0.741 |  | 1.22  (0.64-2.34) | **1.39**  **(1.07-1.80)** | 0.506 |

Adjusted for age, gender, marital status, ethnicity, index of deprivation, MMSE score, HoNOS scores (agitation, psychosis, non-accidental self-injury, problem-drinking or drug taking, depressed mood, physical illness or disability, activities of daily living, living conditions, occupational / recreational activities, social relationships), and hospitalisation in the year prior to dementia diagnosis (Model 3)

**Bold** p<0.05

***Italics*** 0.05<p<0.10
